# HMGB1 promotes differentiation syndrome by inducing hyperinflammation via MEK/ERK signaling in acute promyelocytic leukemia cells

**DOI:** 10.18632/oncotarget.15432

**Published:** 2017-02-17

**Authors:** Lanlan Tang, Wenwen Chai, Fanghua Ye, Yan Yu, Lizhi Cao, Minghua Yang, Min Xie, Liangchun Yang

**Affiliations:** ^1^ Department of Pediatrics, Xiangya Hospital, Central South University, Changsha, Hunan 410008, People's Republic of China; ^2^ Department of Nuclear Medicine, Hunan Cancer Hospital and The Affiliated Cancer Hospital of Xiangya School of Medicine, Central South University, Changsha, Hunan 410008, People's Republic of China

**Keywords:** HMGB1, differentiation syndrome, cytokines, adhesive molecule, MEK/ERK

## Abstract

Differentiation therapy based on all-trans-retinoic acid (ATRA) and arsenic trioxide (ATO) for the treatment of acute promyelocytic leukemia (APL) is complicated by the development of differentiation syndrome (DS), which can be fatal. We examined the role of HMGB1 (high-mobility group box 1) in DS using both *in vitro* and *in vivo* models. HMGB1 and the pro-inflammatory cytokines IL-1β and TNF-α were gradually released from NB4 and HL-60 cells treated with ATRA and/or ATO. Similarly, higher serum HMGB1 levels positively correlated with the clinical status of DS patients. Exogenous HMGB1 promoted rapid release of IL-1β and TNF-α as well as elevated expression of ICAM-1, without altering cell differentiation. Exogenous HMGB1 also enhanced pulmonary infiltration and up-regulated ICAM-1 expression in the ATRA-treated DS mouse. Pharmacological inhibition or depletion of MEK1/2 reduced the cytokine levels and suppressed expression of ICAM-1 and the adhesion of HMGB1-treated NB4 cells to endothelial cells, implicating MEK/ERK signaling in the response to HMGB1 during DS. Treatment with a HMGB1-neutralizing antibody reduced secretion of TNF-α and IL-1β, arrested the elevation of ICAM-1 and blunted the activation of ERK1/2 in ATRA-induced NB4 cells. The HMGB1-neutralizing antibody also decreased ICAM-1 expression and reduced mortality in ATRA-treated DS model mice. These findings demonstrate that released HMGB1 is central to DS, and that targeting HMGB1 may be of therapeutic value in the treatment of DS.

## INTRODUCTION

Acute promyelocytic leukemia (APL) is characterized by a reciprocal balanced translocation between chromosomes 15 and 17 [t (15; 17)] leading to a fusion between the promyelocytic leukemia (PML) and the retinoic acid receptor-α (RARα) gene [1]. Morphologically, APL is categorized as AML-M3 based on the French-American-British (FAB) classification scheme. All-trans retinoic acid (ATRA) and arsenic trioxide (ATO) are used for APL therapy since they degrade the PML-RARα oncoprotein and differentiate the leukemic cells resulting in clinical remission of APL patients [2]. However, long-term exposure of ATRA and ATO results in hyper-inflammation and development of the differentiation syndrome (DS) [3, 4].

The DS involves the cytokine storm that coincides with the differentiation of blasts [3]. Yet the underlying pathogenic mechanisms for DS, which was previously called the retinoic acid syndrome that can be fatal to APL patients are unknown. The classic symptoms of DS include respiratory distress, unexplained fever, weight gain, interstitial pulmonary infiltration, pleural or pericardial effusion, hypotension and acute renal failure [5]. Complications that occurred during induction therapy with ATRA and/or ATO when leukemic blasts were abundant were rare during consolidation or maintenance therapy [6, 7]. Either alone or in combination with cytotoxic drugs, induction therapy with ATRA or ATO induced DS in 2-30% of APL patients [8]. Treatment with corticosteroids and close monitoring of symptoms has reduced DS associated mortality from historical high of 30% to about 2–10% [9]. Therefore, mechanistic understanding of the differentiation-related hyper-inflammation is necessary to identify novel therapeutic targets for DS.

As a highly conserved nuclear protein, HMGB1 (high-mobility group box 1) is a highly conserved nuclear protein that acts as a chromatin-binding factor for bending DNA and promoting access to transcriptional protein assemblies on specific DNA targets [10]. Additionally, it functions as a prototypic damage-associated molecular pattern (DAMP) molecule that is released from various cancer cells after chemotherapy or radiotherapy and is implicated in many cancer associated inflammation disorders [11]. The release of HMGB1 was identified as a trigger for acute anti-neoplastic inflammation that was initiated against tumor cells during chemotherapy [12]. Tumor-derived HMGB1 inhibited the cytotoxic anti-tumor CD8+ cells by potentiating regulatory IL-10-producing T cells [13]. Also, exogenous HMGB1 positively correlated with the clinical status in childhood leukemia and stimulated leukemic cells to secrete TNF-α [14]. However, the role of HMGB1 including its impact on hyper-inflammation during the development of DS in APL patients is unknown.

Therefore, in this study we explored the relationship between exogenous HMGB1 and the clinical status of DS patients by examining its effects on NB4 cell differentiation, pro-inflammatory cytokine secretion and expression of cell adhesive molecules. Further, we investigated the signaling pathway activated by HMGB1 that regulates the pathological mechanisms in DS using both the *in vitro* and *in vivo* DS mouse model.

## RESULTS

### HMGB1 release and correlation with clinical stage of DS patients

**Figure 1:**
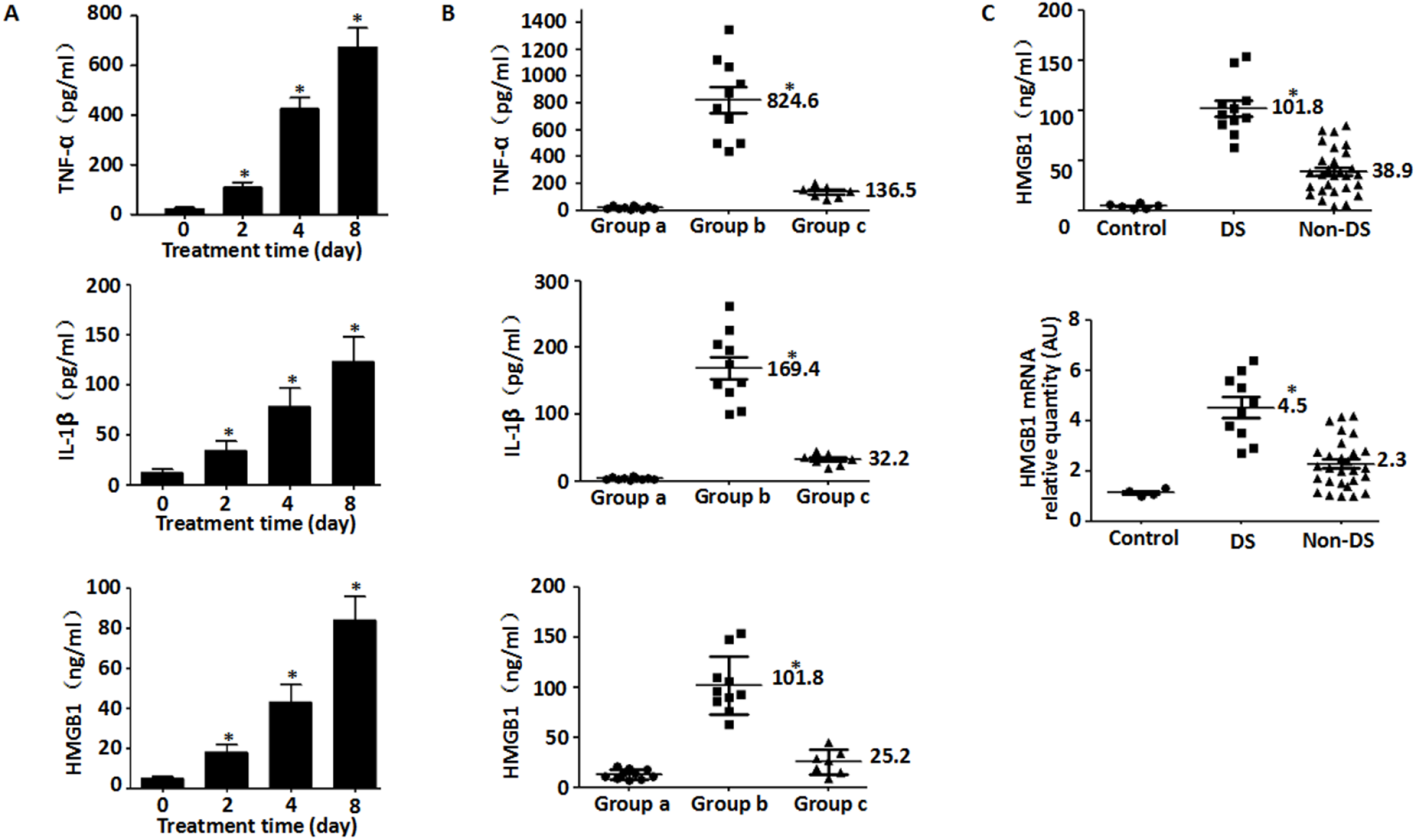
HMGB1 and pro-inflammtory cytokines are released from cells during DS **A**. Quantification of serum TNF-α, IL-1β and HMGB1 levels after ATRA treatment (25 mg/m^2^/day) in one patient for 0-8 day by ELISA (n=3, *P<0.05 versus untreated group); **B**. Quantification of serum HMGB1 and cytokines after ATRA treatment (25 mg/m^2^/day) in 10 DS patients with different clinical status. The serum amounts of TNF-α, IL-1β and HMGB1 in the DS patients at different time points during DS was analyzed by ELISA. Each dot represents the level of TNF-α, IL-1β or HMGB1 in each sample. Group a represents time point before ATRA treatment; Group b represents time point when DS developed; Group c represents time point with DS complete remission (n=3, *P<0.05 versus group a/c). **C**. Quantification of HMGB1 protein and mRNA in the sera of DS and non-DS patients after ATRA treatment. A total of 38 patients with newly diagnosed APL received ATRA were analyzed. The serum samples of DS patients were collected range from day 3 to 16. If patients didn't demonstrate DS in 2 weeks, serum samples of non-DS patients were collected at day 14. HMGB1 protein and mRNA in sera were detected by ELISA and real-time quantitative PCR, respectively. Each dot represents HMGB1 levels in each sample. Control, normal healthy subjects; DS, patients with differentiation syndrome; non-DS, patients without differentiation syndrome (n=3, *P<0.05 versus non-DS group).

During induction treatment for APL, DS manifests between 2 to 46 days with the predominant symptoms being fever, respiratory failure and fluid retention resulting in weight gain [3, 4]. The criteria for definitive DS diagnosis included appearance of three or more symptoms and signs [15]. The most severe clinical outcome of DS during ATRA treatment of APL is hyper-inflammation that involves excessive cytokine secretions and induction of cell surface adhesive molecules [3]. Therefore, to study DS and the causative factors, we enrolled 38 patients from January 2012 to December 2015 that were newly diagnosed with APL and aged between 1-13 years. These patients received 25 mg/m^2^/day ATRA plus cytarabine and daunorubicin chemotherapy as induction treatment. Firstly, we quantified the serum levels of IL-1β, TNF-α and HMGB1 from 1 case of newly diagnosed APL patient developed DS on the eighth day after ATRA treatment using ELISA. We observed a gradual increase suggesting that HMGB1 was linked to inflammatory response during induction treatment of APL (Figure 1A).

Further, we observed that ten of the 38 patients developed DS, of which 4 were severe [≥4 symptoms of DS] and 6 were moderate [3 symptoms of DS]. The median time for development of DS from the start of ATRA administration was 9 days (range: 3-16). Eight of the ten DS patients (80%) achieved remission under close observation and prompt administration of corticosteroids, whereas two others (20%) died. To investigate the plausible reasons for DS in these patients, cytokine levels were measured at various clinical stages. Our data suggested that the serum concentrations of IL-1β, TNF-α and HMGB1 were elevated in DS patients compared to primary and DS remission stages (Figure 1B) and hence were closely linked to the clinical status of the disease.

To further characterize the role of HMGB1 in DS, its release was evaluated in the 38 APL patients after chemotherapy using ELISA. As shown in Figure 1C, the serum levels of HMGB1 protein and mRNA were elevated in DS patients compared to normal healthy controls and non-DS patients. Moreover, leukocytosis correlated with higher HMGB1 secretion at diagnosis (a median WBC count of 8.6×10^9^/l). These data suggested that HMGB1 was a prognostic indicator for DS (Table 1).

**Table 1:** Clinical variables of DS patients*

Variables at diagnosis	DS group	Non-DS group	P-value^a^
HMGB1 (ng/ml)	94.2 (63-154.1)^b^	35.7 (10.5-79.4)	0.000
WBCs (×10^9^/l)	8.6 (1.5-54.7)	3.4 (0.5-22.6)	0.021
Hb (g/l)	76 (29-114)	81.5 (65-95)	0.289
Platelet (×10^9^/l)	33(5-86)	29.5 (5-321)	0.829
Age (years)	8.5(3-13)	6(1-13)	0.147

DS, differentiation syndrome; WBCs, white blood cells; Hb, hemoglobin; *patients having DS (≥3 symptoms of DS); ^a^P-value in Mann-Whitney U test; ^b^median values (minimum-maximum).

### Induction therapeutic agents, ATRA and ATO promote HMGB1 and cytokine release in human myeloid cells

**Figure 2:**
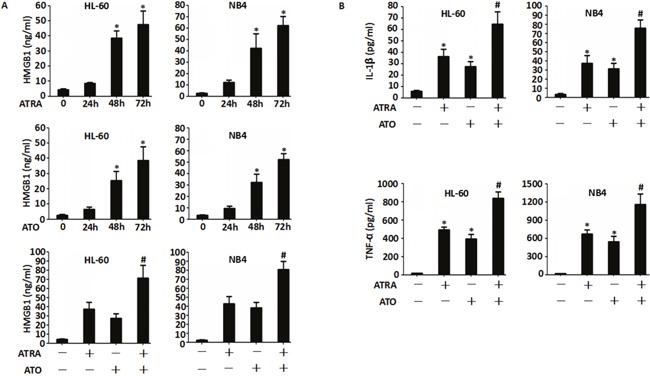
All-trans-retinoic acid (ATRA) and arsenic trioxide (ATO) promote release of HMGB1 and cytokine release in human myeloid cells **A**. HL-60 and NB4 cells were treated with either ATRA (1 μM) or ATO (5 mM) for 24-72 h or ATRA plus ATO for 48 h and the amount of HMGB1 released into the supernatant was analyzed by ELISA. (n=3, *P<0.05 versus untreated group; #*P*<0.05 versus ATRA or HMGB1 group). **B**. HL-60 and NB4 cells were treated with ATRA (1 μM) or/and ATO (5 mM) for 48 h and the levels of TNF-α and IL-1β released into the supernatant were analyzed by ELISA. (n=3, *P<0.05 versus untreated group; #*P*<0.05 versus ATRA or HMGB1 group).

To gain investigate the mechanisms underlying DS, we treated human myeloid cells, HL-60 and NB4 with ATRA and/or ATO for 24–72 h and quantified the supernatant concentrations of HMGB1, IL-1β and TNF-α by ELISA. We observed that HMGB1 levels gradually increased, especially in the ATRA plus ATO group compared to non-treated cells (Figure 2A). Further, ATRA and/or ATO treatments resulted in elevated levels of IL-1β and TNF-α in the supernatant of both the cell types, 48 h after treatment (Figure 2B). This suggested that the release of HMGB1 and the cytokines was a universal event upon treatment with ATRA and/or ATO.

### Exogenous HMGB1 has no effect on cell differentiation

**Figure 3:**
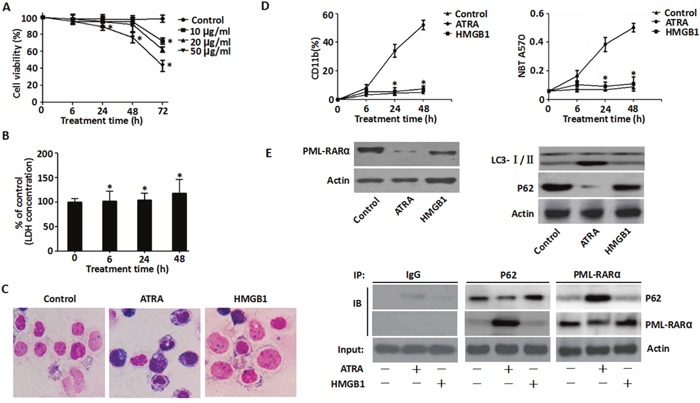
Effects of exogenous HMGB1 on ATRA-induced differentiation **A**. Viability of NB4 cells that were treated with exogenous HMGB1 (10, 20 & 50 μg/ml) for 6-72 h was determined by the CCK-8 assay. Viability of control cells (DMSO) was set as 100%. (n=3, *P <0.05 versus control group). **B**. LDH released by NB4 cells that were treated with HMGB1 (10 μg/ml) for 6-48 h was detected by LDH assay kit and expressed as percentage of control (n=3, *P>0.05 versus untreated group). **C**. Morphological features of NB4 cells that were treated with ATRA (1 μM) or HMGB1 (1 μg/ml) was determined by Wright-Giemsa staining and visualized by light microscopy (100X magnification). **D**. Differentiation of NB4 cells treated with ATRA (1 μM) or HMGB1 (10 μg/ml) for 6-48 h was examined by NBT reduction and surface CD11b expression in comparison to the control (DMSO) (n=3, *P<0.05 versus ATRA group). **E**. Expression of PML-RARα, P62 and LC3-I/II in NB4 cells treated with ATRA (1 μM) or HMGB1 (10 μg/ml) for 48 h was assayed by western blotting and co-immunoprecipitation in comparison to control (DMSO).

To determine if DS was induced by the exogenous HMGB1, the effect of recombinant HMGB1 protein on cell viability was determined at various concentrations (10, 20 & 50 μg/ml) for 6-72 h (Figure 3A). At 10 and 20 μg/ml, HMGB1 did not inhibit growth between 6-48 h (inhibitory percentage <10%). However, treatment with 50 μg/ml HMGB1 resulted in significant growth inhibition between 6-72 h (Figure 3A). Further, as shown in Figure 3B, we observed time-dependent increase in LDH in the supernatant of NB4 cells treated with 10 μg/ml HMGB1 suggesting compromised cell membrane integrity in accordance with previous studies [18, 32].

To determine the role of exogenous HMGB1 in differentiation of APL cells, the morphology of recombinant HMGB1 protein-treated NB4 cells was assessed. Aggregation of NB4 cells with irregular nuclei was observed at 48 h after incubation with ATRA, but no morphological differences were observed in cells treated with HMGB1 (Figure 3C). Further, enhanced expression of myeloid differentiation cell surface marker CD11b and the functional differentiation as determined by NBT reduction was observed only upon ATRA treatment and HMGB1 treatment had no effect (Figure 3D).

The direct molecular target of ATRA in human myeloid cells is the PML-RARα oncoprotein that mediates differentiation. Previously, we demonstrated that up-regulated endogenous HMGB1 promoted autophagy and induced NB4 cell differentiation via ubiquitin-binding adaptor protein p62/SQSTM1-mediated degradation of PML-RARα oncoprotein [16]. However, in this study, the exogenous treatment of cells with the recombinant HMGB1 protein failed to induce degradation of PML-RARα and conversion of LC3-I to LC3-II in comparison to ATRA treatment (Figure 3E). Further, P62 and PML-RARα did not co-IP in cells treated with exogenous HMGB1 compared to ATRA (Figure 3E). This suggested that exogenous HMGB1 did not induce cell differentiation.

### HMGB1 induced cytokine secretion, adhesive molecule expression and enhanced endothelial adhesion

**Figure 4:**
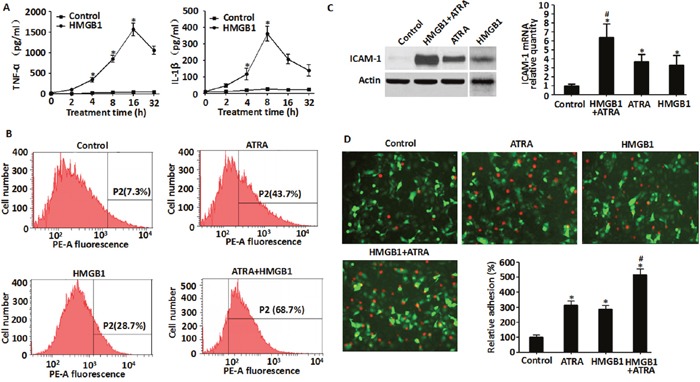
Exogenous HMGB1 induced cytokine secretion, up-regulated expression of ICAM-1 and enhanced endothelial adhesion in NB4 cells **A**. Levels of TNF-α and IL-1β secreted by NB4 cells that were treated with HMGB1 (10 μg/ml) for 2-32 h were detected by ELISA. (n=3, *P<0.05 versus control group). **B**. and **C**. Levels of ICAM-1 expressed in NB4 cells that were treated with ATRA (1 μM) for 48 h, HMGB1 (10 μg/ml) for 8 h or ATRA (1 μM) for 48 h followed by HMGB1 (10 μg/ml) for 8 h were determined by flow cytometry, western blot and qRT-PCR. ICAM-1 levels were expressed as a percentage of control (DMSO) (n=3, *P<0.05 versus control group; #*P*<0.05 versus ATRA or HMGB1 group). **D**. Determination of cell adhesiveness of NB4 cells after treatment with ATRA (1 μM) for 48 h, HMGB1 (10 μg/ml) for 8 h or ATRA (1 μM) for 48 h followed by HMGB1 (10 μg/ml) for 8 h. Towards this, NB4 cells were fluorescently tagged by CM-Dil. Then, the fluorescent NB4 cells were co-incubated with EA.hy926 endothelial cells (transfected with pEGFP-N1 vector) and observed microscopically (100X magnification) and quantified fluorometrically. The data were expressed as a percentage of the DMSO control (n=3, *P<0.05 versus control group; #*P*<0.05 versus ATRA or HMGB1 group).

Mounting evidence suggests that hyper-inflammation is critical for the development of DS [3, 4]. To determine if exogenous HMGB1 directly induced inflammatory response in NB4 cells, we observed its effects on pro-inflammatory molecules involved in DS, including TNF-α, IL-1β and ICAM-1. Treatment of recombinant HMGB1 (10 μg/ml) protein resulted in the release of TNF-α (from 2 h to 16 h) and IL-1β (from 2 h to 8 h) in a time-dependent manner (Figure 4A). Moreover, ICAM-1 expression was significantly elevated with ATRA and/or recombinant HMGB1 protein treatment based on FACS, western blot and qRT-PCR data (Figure 4B & 4C). Combination of ATRA and HMGB1 lead to greater ICAM-1 expression than ATRA alone (Figure 4B & 4C). These data suggested a potential pro-inflammatory role for exogenous HMGB1 in ATRA-induced differentiation. Endothelial adhesion of activated leukocytes is a critical step during inflammation [17]. Therefore, when NB4 cells treated with ATRA and HMGB1 were co-incubated with EA.hy926 endothelial cells, increased endothelial adhesion was observed (Figure 4D) suggesting that HMGB1 was involved in inflammation.

### Exogenous HMGB1 promotes lung infiltration in the DS model

**Figure 5:**
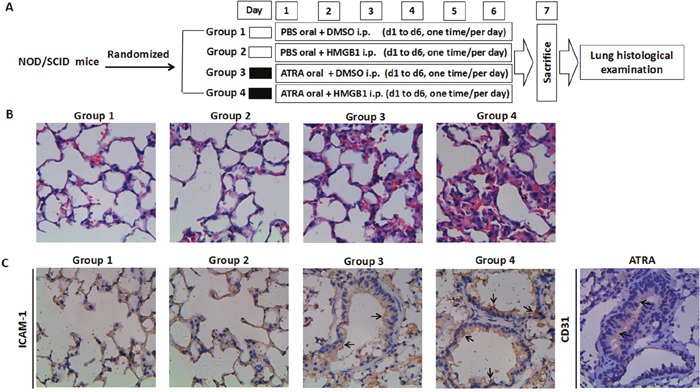
Effects of HMGB1 on pulmonary infiltration in mice **A**. Experimental scheme shows twenty-four mice divided into four groups that received different treatments. Note: i.p. = intraperitoneal injection. **B**. Hematoxylin & Eosin staining of lung tissue sections. After 6-days of treatments, the mice from the four groups were sacrificed, the lungs were removed and tissue sections were prepared as described in the methods. After staining with hematoxylin & eosin, the specimens were examined microscopically under high power field (400X magnification). **C**. Immunohistochemistry of ICAM-1 in lung tissue sections of mice from the four treatments was performed as described in the methods. The specimens were examined microscopically under 400X magnification. The lung vascular endothelial cells were marked by CD31 antibody. The arrows indicate positive immunoreactivity of ICAM-1 and/or CD31.

Similar to a previous study [18], we created a NOD/SCID mouse model of DS by injecting ATRA-treated NB4 cells followed by continuous administration of ATRA. The study design is summarized in Figure 5A. We evaluated the effect of HMGB1 on the histology of lung based on hematoxylin & eosin (HE) staining. Mice that received a tail vein injection of ATRA-untreated NB4 cells followed by a 6-day oral administration of PBS and an abdominal injection of DMSO or recombinant HMGB1 protein did not show any cellular infiltration in the lungs (Figure 5B, groups 1 & 2). However, mice that received ATRA-treated NB4 and subsequent six-day oral administration of ATRA and abdominal injection of DMSO (HMGB1's control) demonstrated severe cellular infiltration, widened lung intervals, highly congested pulmonary interstitial space and fractured alveolar walls (Figure 5B, group 3). Moreover, the DS model mice that were injected abdominally with recombinant HMGB1 protein showed significant changes in lung tissue compared to group 3 (Figure 5B, group 4). Furthermore, immunohistochemistry revealed that ICAM-1 was highly expressed in the lung vascular endothelial cells (CD31 positive) of ATRA-treated DS model mice (Figure 5C, group 3). Since co-treatment of exogenous HMGB1 and ATRA (group 4) significantly increased the expression of ICAM-1 compared to group 3 (Figure 5C), we concluded that HMGB1 effectively promoted lung infiltration in the DS model.

### Requirement of MEK/ERK pathway for HMGB1-mediated cytokine secretion and ICAM-1 elevation

**Figure 6:**
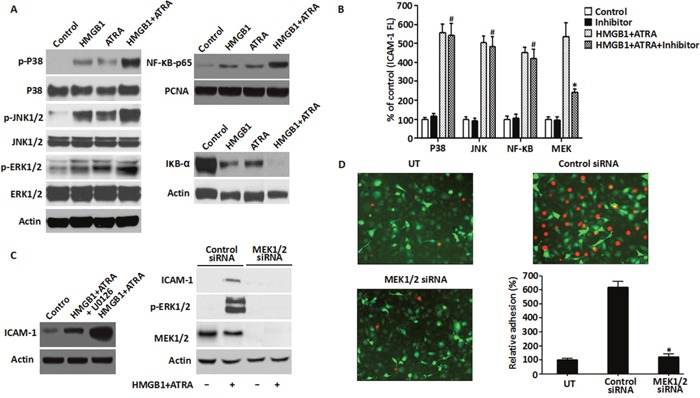
Role of MEK/ERK pathway in HMGB1-mediated cytokine secretion and ICAM-1 elevation **A**. The MAPK signaling pathway was analyzed in NB4 cells that were treated with ATRA (1 μM) for 48 h or/and HMGB1 (10 μg/ml) for 8 h by western blot analysis of phosphorylated and non-phosphorylated p38, Jnk and Erk kinases. Also, NF-kB pathway was analyzed by detecting the levels of p65 and IkB-α proteins for the same treatments described above along with PCNA. Actin was used as control. **B**. ICAM-1 levels were determined by FACS analysis of NB4 cells that were treated with ATRA (1 μM) for 48 h followed by HMGB1 (10 μg/ml) for 8 h with or without pre-treatment of the p38, Jnk, NF-kB or MEK inhibitors for 30 min (n=3, *P<0.05 versus MEK ATRA+HMGB1 group; #*P*>0.05 versus P38, JNK or NF-κB ATRA+HMGB1 group). **C**. The requirement for MEK/ERK signaling in DS was analyzed by detecting the levels of ICAM-1, MEK1/2 and p-ERK by western blot in NB4 cells that after pre-treatment with U0126 (20 μM) for 30 min or transfection with MEK1/2 siRNA for 48 h were induced by ATRA (1μM) for 48h followed by HMGB1 (10 μg/ml) for 8 h. **D**. Adhesion property of NB4 cells that were transfected with MEK1/2 or control siRNA for 48 h and treated with ATRA (1 μM) for 48 h followed by HMGB1 (10 μg/ml) for 8 h was analyzed as described in the methods. Briefly, the treated cells were fluorescence-labeled with CM-Dil and then co-incubated with EA.hy926 endothelial cells (transfected with pEGFP-N1 vector) to monitor cell-cell adhesion. Fluorescent NB4 cells adherent on endothelial cells were observed microscopically and quantified fluorometrically (100X magnification). The data were expressed as percentages relative to the control group (n=3, *P<0.05 versus control siRNA group).

The cytokines and ICAM-1 are up-regulated by a variety of intracellular signaling pathways, including mitogen-activated protein kinases (MAPKs) and nuclear transcriptional factors-κB (NF-κB) [19]. To determine if exogenous HMGB1 directly regulated these pathways, we analyzed the activation of ERK, JNK, p38 MAPKs and NF-κB in ATRA and/or HMGB1-treated NB4 cells. We observed that ERK1/2, JNK1/2, p38 and NF-κB (p65) were activated as suggested by their phosphorylation in response to treatment with ATRA and/or recombinant HMGB1 protein (Figure 6A). Further, combination of ATRA and exogenous HMGB1 showed greater activation compared to ATRA alone (Figure 6A). These results suggested that ERK1/2, JNK1/2, p38 and NF-κB (p65) were primary candidates for exogenous HMGB1-mediated inflammation. To further confirm this, ICAM-1 expression was analyzed in HMGB1 and ATRA-treated NB4 cells in presence of MEK, JNK, p38 and NF-κB inhibitors were used. Only MEK inhibitor partially inhibited ICAM-1 elevation (Figure 6B & 5C). Furthermore, transfection of target-specific shRNAs against MEK1/2 that depleted MEK1/2 expression inhibited both ICAM-1 and p-ERK1/2 (Figure 6C). This suggested that MEK/ERK was necessary for exogenous HMGB1-mediated ICAM-1 elevation in ATRA-induced NB4 cells.

To further characterize the role of the MEK/ERK pathway in exogenous HMGB1-mediated inflammation, cytokine secretion and cell adhesion molecules were analyzed in ATRA and HMGB1-treated cells in presence of either MEK1/2 inhibitor (U0126) or MEK1/2 depletion. U0126 significantly diminished the secretion of TNF-α and IL-1β upon HMGB1 and ATRA co-induction (Table 2). Moreover, knockdown of MEK1/2 attenuated the adhesion of exogenous HMGB1 and ATRA co-treated NB4 cells in the endothelial adhesion assay (Figure 6D). This suggested that HMGB1 mediated inflammation through the MEK/ERK signaling pathway in the ATRA-induced NB4 cells.

**Table 2:** Effects of U0126 on the secretion of cytokines in HMGB1-mediated NB4 cells

	Time	Control	U0126	HMGB1+ATRA	HMGB1+ATRA+U0126
IL-1β (pg/ml)	24h	2.8±0.6	2.4±0.7	6.9±0.8	3.0±1.2
	48h	5.9±0.7	5.7±0.8	25.3±1.2*	9.2±0.7**
	72h	6.5±1.1	8.2±1.1	72.6±3.0 *	30.5±2.9**
TNF-α (pg/ml)	24h	11.3±2.3	12.8±2.7	42.1±8.4	12.9±2.3
	48h	21.1±2.0	20.2±1.4	128.9±18.0*	71.0±14.8**
	72h	50.9±7.5	43.3±7.6	539.7±51.1*	293.2±44.7**

Note: *P <0.01, vs control group; **P<0.05, vs HMGB1 group

### HMGB1 neutralizing antibody reduced the inflammatory response to ATRA treatment

**Figure 7:**
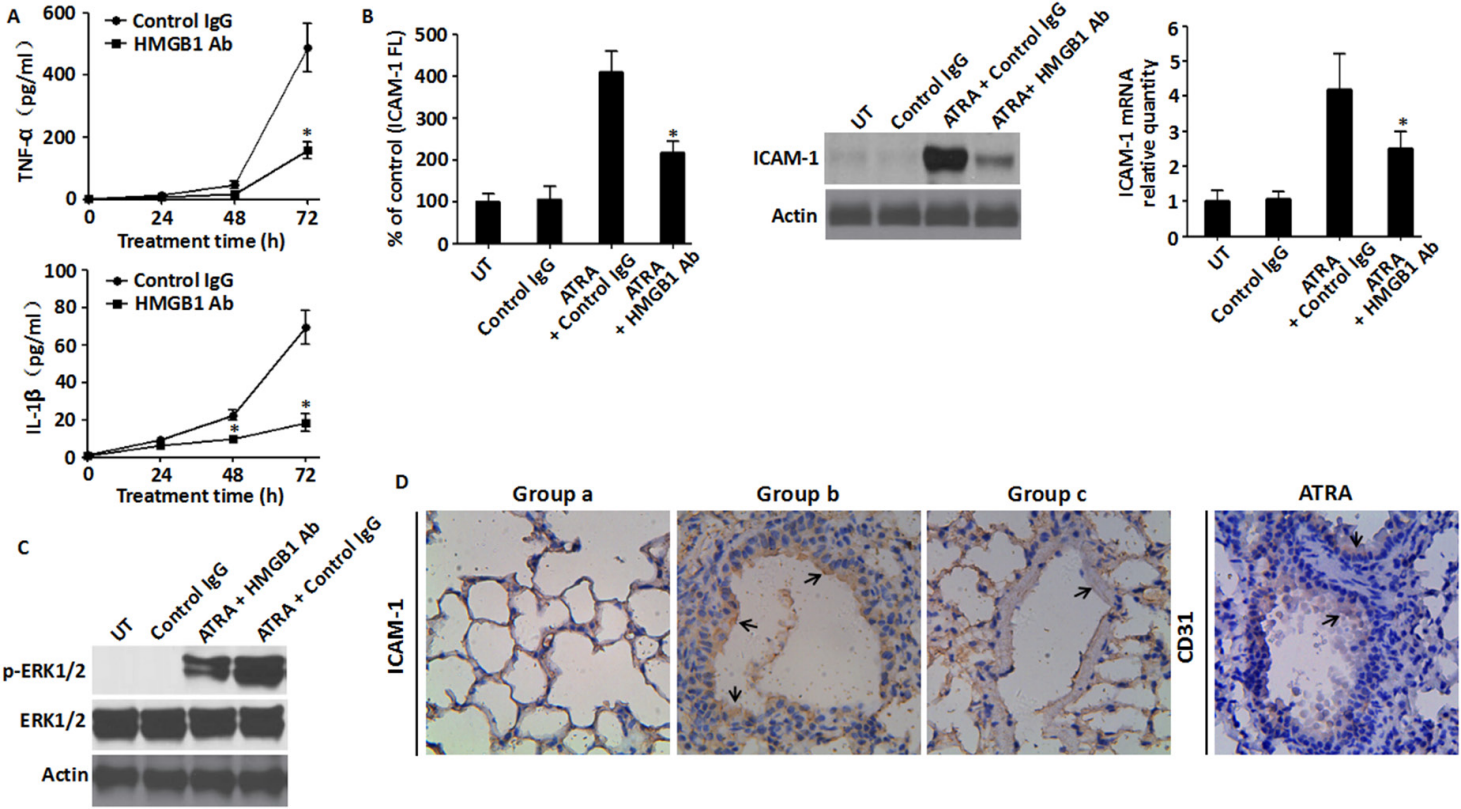
Inhibition of HMGB1 level blunted HMGB1-mediated inflammatory response **A**. The levels of TNF-α and IL-1β in the supernatant of NB4 cells that were treated with ATRA (1 μM) for 24-72 h with or without HMGB1-neutralizing antibody (10 μg/ml) were analyzed by ELISA (n=3, *P<0.05 versus control IgG group). **B**. ICAM-1 levels in NB4 cells that were treated with ATRA (1 μM) for 48 h with or without co-incubation with HMGB1-neutralizing antibody (10 μg/ml) was determined by flow cytometry (left, surface expression), western blot (center, total protein) and qRT-PCR (right, mRNA). Incremental production of ICAM-1 was expressed as a percentage of control (n=3, *P<0.05 versus ATRA + control IgG group). **C**. The activation of the MEK/ERK signaling pathway was analyzed in NB4 cells that were treated with ATRA (1μM) for 48 h with or without co-incubation with HMGB1-neutralizing antibody (10 μg/ml) by western blot analysis of ERK1/2 and p-ERK1/2. **D**. Immunohistochemistry of ICAM-1. Lung tissue sections were prepared from the mice that were sacrificed 6-days after treatment and immunohistochemical staining of ICAM-1 was performed as described in the methods. The lung vascular endothelial cells were marked by the CD31 antibody with the arrows indicating positive immunoreactivity of ICAM-1 and CD31. Group a, non-DS model receiving DMSO; Group b: DS model receiving DMSO; Group c: DS model receiving HMGB1-neutralizing antibody. (400X magnification).

To further find conclusive evidence regarding the regulation of HMGB1 in pro-inflammatory function after chemotherapy, ATRA-induced NB4 cells that were incubated with 10 μg/ml neutralizing antibody against HMGB1 [20] showed partial reduction of TNF-α and IL-1β secretion compared to the negative control (Figure 7A). In addition, treatment of HMGB1-neutralizing antibody partially inhibited ICAM-1 elevation (Figure 7B) and reduced ERK1/2 phosphorylation compared to the ATRA-treated group (Figure 7C). Moreover, the ATRA-treated DS mouse model with an abdominal injection of HMGB1-neutralizing antibody showed a significant decrease of ICAM-1 expression (Figure 7D, group c) and mice death (Supplementary Figure 2). These data conclusively supported the pro-inflammatory function of exogenous HMGB1 in ATRA chemotherapy that lead to DS.

## DISCUSSION

The pathogenesis of DS is not well understood. Mounting evidence suggests that sustained hyperinflammation is critical for the development of DS [3, 4]. Emigration of ATRA-treated APL cells into lungs and other tissues recapitulates the molecular events during inflammation and immune responses [21]. The major molecular mediators of inflammation during ATRA-induced APL are pro-inflammatory cytokines and ICAM-1 [20, 22]. ATRA induces release of early pro-inflammatory cytokines such as TNF-α and IL-1β [22, 23], which promote endothelial adhesion in NB4 cells [24, 25]. In comparison to IL-1β and TNF-α, exogenous HMGB1 is released in a delayed manner and functions as a late inflammation mediator. As a potential therap*e*utic target, it provides a broader time window for clinical interventions [26]. In our study, we demonstrated elevated TNF-α and IL-1β levels in the cell supernatant and patient sera after chemotherapy. Also, similar to the cytokine secretions, ATRA induced HMGB1 release into the cell supernatants and patient sera. Therefore, the expression of HMGB1 positively correlated with the clinical status of DS suggesting that it is involved in the mechanism leading to DS.

HMGB1 contributes to the pathogenesis of various human diseases including leukemia and maybe relevant for understanding the mechanisms underlying DS and its therapy [27, 28]. Previous studies have shown that exogenous HMGB1 of levels reach as high as 200 ng/ml during anti-neoplastic immunity of leukemia chemotherapy and induce TNF-α and IL-1β secretion [14, 29]. However, at lower doses, HMGB1 failed to trigger inflammasome activation [29]. Consistent with previous studies, 10 μg/ml HMGB1 induced a time-dependent increase in TNF-α and IL-1β secretion. Moreover, HMGB1 dosing had no obvious effects on cell differentiation suggesting that suppressed release of HMGB1 may be a novel way of blunting differentiation-associated hyper-inflammation.

In addition to inducing the secretion of cytokines, HMGB1 promoted ATRA-induced ICAM-1 expression. Widely expressed at basal levels on the surface of endothelial cells, leukocytes and the APL cell line NB4, ICAM-1 is up-regulated by pro-inflammatory cytokines and ATRA [30, 31]. Association between ICAM-1 polymorphism and DS exists in ATRA-treated patients [32]. This is further reiterated by the fact that knockout of the ICAM-1 gene abolished inflammation-related syndrome in mice [33]. Furthermore, the interaction between endothelial cells and the extracellular matrix during leukocytic transmigration from the bloodstream into the tissues is critical for inflammation during the development of DS [24] and ICAM-1 is a principal mediator of leukocyte adhesion [34]. In this study, ATRA and/or exogenous HMGB1 significantly enhanced ICAM-1 elevation and endothelial adhesion in the *in vitro* assays as well as in the animal model of the DS [18]. Most DS patients manifest pulmonary changes due to leukemic pulmonary infiltration, granulocytic transmigration and endothelial leakage [20]. In our study, co-treatment of HMGB1 led to the classic manifestations of DS, i.e. severe cellular infiltration, widened pulmonary intervals, highly congested pulmonary interstitial space and fractured alveolar walls. Also, high upregulation of ICAM-1 was observed in the alveolar epithelial cells and pulmonary perivascular space. Thus both *in vitro* and *in vivo* data suggested that HMGB1 promoted hyperinflammation during ATRA treatment of APL.

The expression of cytokines and ICAM-1 is regulated by intracellular signaling pathways as MAPKs and NF-κB [35]. The ERK, JNK and p38 MAP kinases participate in cell proliferation, differentiation and inflammation [36]. The ubiquitous pleiotropic transcription factor, NF-κB activation plays vital roles in inflammation, immunity and survival [37]. As a late inflammation mediator, extracellular HMGB1 has been shown to mediate the release of TNF-α, IL-1β and other inflammatory mediators, endothelial cell activation, stromagenesis, recruitment and activation of innate immune cells and maturation of dendritic cells, thereby leading to chronic inflammatory response and activation of protein kinase B (AKT), MAPKs and NF-κB [38]. In the present study, exogenous HMGB1 enhances ATRA-induced phosphorylation of ERK, JNK, p38 and NF-κB, thereby implicating the MAPKs and NF-κB in the pro-inflammatory function of HMGB1.

The MEK/ERK pathway is a key diagnostic and therapeutic target for leukemia due to its extensive involvement in cell proliferation, differentiation, survival and apoptosis [39]. Extracellular signal-regulated kinase (MEK) functions as an immediate upstream activator of ERK [40]. It is well established that exogenous HMGB1 induces MEK/ERK activation in immune and cancer cells including leukemic cells [14, 41, 42]. Previously, the MEK/ERK pathway was shown to be essential for ATRA-induced ICAM-1 elevation in NB4 cells [23]. In this study, knockdown or pharmacological inhibition of MEK attenuated HMGB1-mediated ICAM-1 elevation, reduced IL-1β/TNF-α secretion and lowered cell adhesion. This suggested that MEK/ERK signaling was necessary for exogenous HMGB1-mediated inflammatory response. Furthermore, dose dependent treatment with anti-HMGB1 antibody significantly inhibited the secretion of cytokines, expression of cell surface adhesive molecules and activation of ERK1/2. In addition, the HMGB1-neutralizing antibody also decreased ICAM-1 expression and reduced ATRA-treated DS mouse model death further supporting the pro-inflammatory function of exogenous HMGB1 during DS.

In conclusion, HMGB1 plays a key role in the development of DS by inducing inflammation through the MEK/ERK signaling pathway. Therefore, HMGB1 is a potential target for DS therapy.

## MATERIALS AND METHODS

### Reagents and antibodies

RARα (sc-773), MEK1/2 (sc-436), NF-κB-p65 (sc-109), IκB-α (sc-1643), p62 (sc-28359) and actin (sc-47778) antibodies were obtained from Santa Cruz Biotechnology (TX, USA); The antibodies to CD11b (C0551) and ICAM-1 (C2969) were from Sigma(St. Louis, MO, USA); Antibody to LC3 (#NB600-1384) was from Novus (Littleton, CO, USA); The p-ERK1/2 (#9101), ERK1/2 (#4695), p-JNK (#9251), JNK (#9252), p-P38 (#9215), P38 (#9212) and PCNA (#2586) antibodies were from Cell Signaling Technology (MA, USA); ATRA, ATO, DMSO, JNK inhibitor (SP600125), p38 inhibitor (SB203580) and ERK inhibitor (U0126) were from Cell Signaling Technology (MA, USA); p65 inhibitor (BAY11-7802) was from Merck Millipore (Darmstadt, Germany); The recombinant HMGB1 protein was kindly provided by Dr. Rui Kang (University of Pittsburgh, USA).

### Cell culture

NB4 and HL-60 cells (Xiangya School of Medicine Type Culture Collection, China) were cultured in RPMI 1640 medium supplemented with 10% heat-inactivated FBS (Life Technologies, NY, USA) in 5% CO_2_. NB4 is a promyelocytic leukemia cell line, whereas HL-60 represents a discrete stage of differentiation between the late myeloblasts and the promyelocyte [43]. The human endothelial cell line, EA.hy926 (ATCC, VA, USA) was cultured in DMEM (Gibco BRL, NY, USA) supplemented with 10% FBS (Gibco BRL, NY, USA), penivilin (100ug/mL) and streptomycin (100ug/mL). All cells were incubated at 37°C in a humidified atmosphere of 5% CO2 and 95% air.

### Mouse model of DS

Seven- to 8-week male NOD/SCID (non-obese diabetic/severe combined immunodeficient) mice that weighed about 20g were purchased in 2 batches of 24 mice each from the National Rodent Laboratory Animal Resources (Shanghai, China) and maintained at the Shanghai Shilaike Laboratory Animal Co, Ltd. The animal raising and experimental procedures were approved by the Shanghai Animal Welfare & Research Ethics Committee, Science & Technology Committee of Shanghai Municipal Government, China. The animals were raised in pathogen-free conditions with a 12-hour light-dark cycle with water and food provided ad libitum. For each experiment, 24 mice were randomly divided into the following 4 groups: (1) non-DS model receiving DMSO; (2) non-DS model receiving HMGB1; (3) DS model receiving DMSO and (4) DS model receiving HMGB1 treatment (Figure 5A). The DS mice model was established as previously described [18]. Briefly, at Day 1, mice were intravenously injected via a tail vein with 1×10^7^ NB4 cells that were induced by 1mM ATRA. From days 1 to 6, the mice were orally administered with 100 μl ATRA (1 mg/ml) daily. The non-DS model mice received an intravenous injection via tail vein with 1×10^7^ NB4 that were not induced by ATRA and were orally administered with 100 μl of phosphate buffered saline (PBS) every day from days 1 to 6. The HMGB1 or HMGB1-neutralizing antibody (or control DMSO) was administered in the control or model mice by a single intraperitoneal injection of 100 μl PBS containing 10 μg/ml HMGB1 or anti- HMGB1 antibody (or DMSO equivalent) from days 1 to 6. All the mice were sacrificed at day 7 after anesthesia. Their body weights were measured, organs extracted followed by peripheral white blood count and examination of the peripheral blood smear (Supplementary Figure 1). The lung specimens were fixed with 10% formalin followed by cutting 4-μm sections by microtome (Leica, Germany) that were further mounted on slides and stained with hematoxylin and eosin.

### Cell viability assay

Cell viability assays after different treatments were conducted with the Cell Counting Kit-8 (CCK8) kit (Tokyo, Japan) according to the manufacturer's instructions.

### HMGB1, IL-1β and TNF-α quantification by ELISA

The levels of HMGB1, IL-1β and TNF-α in the cell supernatant or patient sera were quantified with the ELISA kits from Shino-Test Corporation (Kanagawa, Japan) or R&D Systems Inc (Minneapolis, MN, USA) according to the manufacturer's instructions.

### Lactate dehydrogenase quantification assay

Cell membrane integrity was determined by assaying the lactate dehydrogenase (LDH) amounts in the supernatant using TOX-7 LDH assay kit (Sigma, MO, USA) [44]. Absorbance at 490nm wavelength was measured using a Wallace 1420 micro-plate reader (PerkinElmer, Waltham, MA, USA). The background optical absorbance at 690 nm was subtracted from the primary measurements for each well. LDH content in medium was calculated by extrapolation from a standard curve and expressed as units/L.

### RNAi and gene transfection

Human MEK1 siRNA (sc-35898: Sense: 5’-GAACCACAUUGAUAUUCUATT-3’; Antisense: 5’-UA GAAUAUCAAUGUGGUUCTT-3’), Human MEK2 siRNA (sc-35905: Sense: 5’-AGAGGCCAAGAGGAUUCCCTT-3’; Antisense: 5’-GGGAAUCCUCUUGGCCUCUTT-3’) and scrambled siRNA were obtained from Santa Cruz Biotechnology (TX, USA). Human MEK1/2 siRNA or pEGFP-N1 control plasmid (a gift from Dr Rui Kang, Hillman Cancer Center, University of Pittsburgh, USA) were performed with FuGENE HD Transfection Reagent (Roche Applied Science, Stockholm, Sweden) according to the manufacturer's instructions.

### Flow cytometry

After culturing under various experimental conditions, the NB4 cells were collected by centrifugation and re-suspended in 50 μl BS-0.5% BSA solution containing adequate concentrations for cell surface adhesion molecules (20 μg/ml ICAM-1) or differentiation markers (10 μg/ml CD11b). Appropriate isotypic antibodies were used as controls. After 30-minute incubation at 4°C in darkness, the cells were washed twice with PBS and resuspended in 600 μl PBSA. Flow cytometry was performed in a BD FACS Calibur. Data acquisition was processed by the Cell Quest software.

### Morphologic observation of differentiation

Cellular morphology was assessed by light microscopy of Wright's-stained cyto-smear preparations as follows: after treatment, NB4 cells were pelleted at 1000 rpm for 5 minutes and the supernatant discarded. Cyto-smear for microscopic examination was prepared by spreading a small drop of cell pellet on a glass microscope slide and air-drying. The smears were subjected to Wright's staining and observed by microscope under high power field. Wright's staining solution solution was from Sigma-Aldrich.

### Nitro blue tetrazolium reduction assay

NB4 cells after specific treatments were centrifuged at 1500 rpm for 5 minutes and re-suspended in equal volumes of RPMI-1640 medium containing 0.2% Nitro Blue Tetrazolium (NBT; Sigma, St.Louis, MO, USA) and 2 μg/ml 12-O-tetradecanoylphorbol-13-acetate (TPA; Sigma, St.Louis, MO, USA) followed by incubation in darkness for 30 minutes at 37°C. The cells were further loaded with 100 μL DMSO into a 96-well plate and after gentle vortexing for 20 minutes, the absorbance of each well was measured at 570 nm in a Thermo Scientific Multiskan Ascent plate reader. The data are representative of three independent experiments.

### Western blot analysis

Proteins in whole-cell lysate or subcellular fractions were resolved on a 10% SDS-PAGE and transferred onto PVDF membrane. After blocking for 3 h at room temperature, the membranes were incubated with various primary antibodies in TBST at 4°C overnight. Further, the membranes were incubated with peroxidase-conjugated secondary antibodies (1:5000) for 1 h at 25°C, followed by visualization of signals based on enhanced chemiluminescence (Pierce Biotechnology, Inc, Rockford, IL, USA.). Membranes were exposed to radiographic film and the expression of targeted proteins was quantified by detecting specific radiographic bands using the BandScan 5.0 system. The experiments were conducted in triplicate.

### Immunoprecipitation

For immunoprecipitation, cells were lysed at 4°C in ice-cold lysis buffer (50 mM Tris-HCl, pH 7.4, containing 150 mM NaCl, 1% NP-40, 0.5% Na-deoxycholate, 0.1% SDS, protease inhibitor cocktail) followed by centrifugation (12000xg,10min) to pellet the cell debris. The protein concentration in the supernatant was determined by bicinchoninic acid (BCA) assay. For immunoprecipitation, equal amounts of protein samples were first pre-cleared with protein A or protein G agarose / sepharose (Santa Cruz, CA, USA) at 4°C for 3h followed by incubation with various specific antibodies (5mg/mL) or control IgG in the presence of protein A or G agarose / sepharose beads for 2h or overnight at 4°C with gentle vortexing. After incubation, the agarose / sepharose beads were thoroughly rinsed with PBS and the proteins eluted by boiling in 2x SDS sample buffer prior to SDS-PAGE.

### Quantitative RT-PCR (qRT-PCR)

Total ribonucleic acid (RNA) was isolated from cells using Trizol reagent (Invitrogen, Carlsbad, CA, USA) according to the manufacturer's protocol. Reverse transcription of 1 μg total RNA was performed using 50 U murine leukemia virus reverse transcriptase, 50 μM random hexamers, and 10 mM deoxynucleoside triphosphates (dNTP) mix (Applied Biosystems, Foster City, CA, USA) in a total reaction volume of 20 μl. Afterwards, qRT-PCR was performed in duplicate with preoptimized primer/probe mixture (TaqMan Gene Expression Assay, Applied Biosystems, Foster City, CA, USA) and TaqMan universal PCR Master Mix (Applied Biosystems, Foster City, CA, USA) on a StepOnePlus Real-Time PCR System (Applied Biosystems, Foster City, CA, USA). The sequences of primers used for qRT-PCR were as follows: (1) ICAM-1 forward 5’-CACAAG CCACGCCTCCCTGAACCTA-3’ and reverse 5’-TGTGGGCCTTTGTGTTTTGATGCTA-3’; (2) HMGB1 forward 5’-TTGGGTCACATGGATTATTAGTGT-3’ and reverse 5’ -CAGGGCATGTGGACAAAAG-3’;(3) β-actin forward 5’-TGGCACCCAGCACAATGAA-3’ and reverse 5’-CTAAGTCATAGTCCGCCTAGAAGCA-3’. The following cycling protocol was used: polymerase activation, 50°C for 2 min, denaturation, 95°C for 10 min, and 35 to 44 cycles of 15 s denaturation at 95°C followed by 1 min of annealing and elongation at 60°C. Data were normalized against the β-actin internal control transcript and were analyzed according to the comparative threshold cycle method. The experiments were performed in triplicates.

### Immunohistochemistry

The lung tissues were fixed in 10% formalin for 24 h followed by dehydration and, paraffin embedding. Then, the paraffin embedded specimens were sliced into 5 μm thick sections by microtome (Leica, Germany) and placed on glass slides. Further, the specimens were deparaffinized, stained with hematoxylin and eosin (H&E) and evaluated under an optical microscope (Olympus BX51, Japan). The pulmonary expression of ICAM-1 and CD31 was determined by immunostaining. After deparaffinization and rehydrating, sections were pressure cooked in antigen retrieval buffer (0.01 M citrate buffer, pH 6.0) for 2 min to unmask antigens. Then, they were incubated with murine anti-rat ICAM-1 and CD31 monoclonal antibodies (1:200, Abcam, MA) at 4°C overnight, followed by biotinylated anti-mouse IgG secondary antibody (ZSGB-bio, China) for 1h at 37°C and streptavidin-HRP (ZSGB-bio, China) for 30 min at 37°C. Further, the HRP substrate, DAB (3, 3-diaminobenzidine; ZSGB-bio, China) was used to develop and visualize the immunostaining, whereas the cell nuclei were counter-stained with hematoxylin. Images were acquired with the Olympus BX51 microscope and the proportion of positive staining cells was determined with the Image-Pro plus 5.1 software.

### Cell adhesion assay

To quantify the cell adhesion ability, the NB4 cells were first loaded in 12-well tissue culture plates at a concentration of 1×10^6^ cells per well in RPMI 1640 medium. After different treatments, the cells were centrifuged at 1000 rpm for 3 minutes and washed twice with PBS. Further, 5×10^6^ cells/mL were resuspended in PBS containing 5 μg/mL CM-Dil (chloromethyl-1,1-dioctadecyl-3,3,3′,3′-tetramethyl- indocarbocyanine perchlorate; Molecular Probes, CA, USA) for fluorescence labeling, incubated at 37°C for 5 minutes followed by additional incubation at 4°C for 15 minutes. The cell pellets were collected by centrifugation, washed twice with PBS and resuspended in RPMI 1640 medium prior to adding the cells onto the endothelial cell culture.

The EA.hy926 endothelial cells were seeded at 2×10^5^ cells/cm^2^ in DMEM onto 6-well tissue culture plates and cultured for 2 days. Then, the culture medium was replaced by RPMI 1640 medium followed by addition of the fluorescence-labeled NB4 cells at a ratio of 1:1. After co-incubation in the RPMI1640 medium for 2 h, the non-adherent NB4 cells were washed off twice with PBS gently so that the endothelial cell monolayer was not disturbed. Fresh RPMI 1640 medium was then added for the remaining cells on the culture plates and the fluorescence-labeled NB4 cells adhering to the endothelial cell monolayer were observed under a Nikon eclipse TE2000-U fluorescence microscope (Tokyo, Japan). For quantification, adherent cells were lysed in 200 μL 0.1 M Tris-HCl containing 0.1% Triton X-100 and centrifuged at 13,000 rpm for 15 minutes. The fluorescent intensity of supernatant was determined at an excitation wavelength of 556nm and an emission wavelength of 573 nm in a LS55 fluorescent spectrometer (PerkinElmer, Waltham, MA, USA) and normalized for endothelial cells.

### Statistical analyses

The data were expressed as mean ±standard error. Statistical significance of differences between treatments was determined by a two-tailed Student's t-test or Mann-Whitney U test. *P*<0.05 was deemed as statistically significant.

## SUPPLEMENTARY FIGURES


