# Supplementary material for: HMGB1 promotes differentiation syndrome by inducing hyperinflammation via MEK/ERK signaling in acute promyelocytic leukemia cells
Source: Oncotarget. 2017 Feb 17;8(16):27314–27. doi: 10.18632/oncotarget.15432 (PMC5432337; doi:10.18632/oncotarget.15432)
Supplement: Supplementary file 1 [file oncotarget-08-27314-s001.pdf]

# HMGB1 promotes differentiation syndrome by inducing hyperinflammation via MEK/ERK signaling in acute promyelocytic leukemia cells

## SUPPLEMENTARY FIGURES

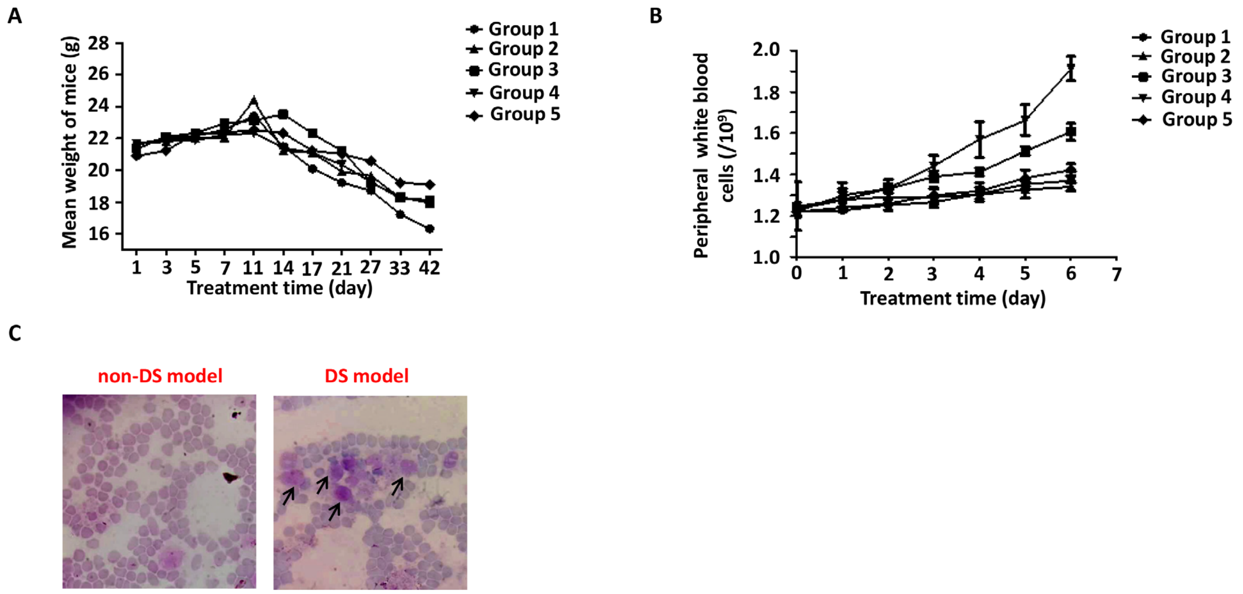


**Supplementary Figure 1: Characterization of DS model mice.** NOD/SCID mice were intravenously injected via a tail vein with NB4 or ATRA-induced NB4 cells, and then were orally administered daily PBS, DMSO, ATRA or/and HMGB1-neutralizing antibody, respectively, for 6 days. **A.** The weight and **B.** peripheral white blood cell count were examined every several days. Group 1: non-DS model receiving DMSO; Group 2: non-DS model receiving HMGB1; Group 3: DS model receiving DMSO; Group 4: DS model receiving HMGB1 treatment; Group 5: DS model receiving HMGB1-neutralizing antibody treatment. Arrows indicate leukemia cells. **C.** Representative peripheral blood smear comparing the non-DS and DS models were examined on day 7. DS model: mice that were intravenously injected via a tail vein with ATRA-induced NB4 cells on day 1 and further orally administered with a daily dose of ATRA (1 mg/ml, 100 µl) for 6 days. Non-DS mode: mice that were intravenously injected via a tail vein with NB4 cells on day 1 and further orally administered daily with PBS (100 µl) for 6 days. Arrows indicate infiltrated cells.

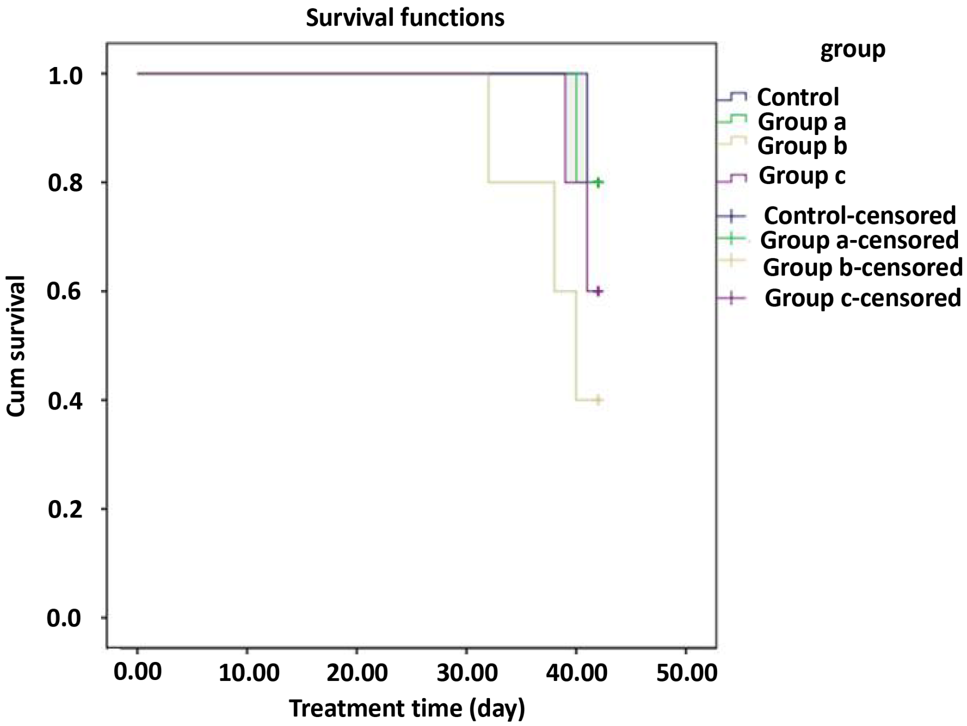


**Supplementary Figure 2: The Kaplan-Meier survival curve of DS model NOD/SCID mice after different treatments.** NOD/SCID mice were intravenously injected via a tail vein with NB4 or ATRA-induced NB4 cells and further orally administered with daily doses of either PBS, DMSO, ATRA or/and HMGB1-neutralizing antibody for 6 days respectively. After 6-day treatments, all mice were fed normal food and the 42-day survival time of mice observed. Control, NOD/SCID mice with no treatment. Group a, non-DS model receiving DMSO; Group b: DS model receiving DMSO; Group c: DS model receiving HMGB1-neutralizing antibody.
